# Feeding containing the aerial part of *Scutellaria baicalensis* promotes the growth and nutritive value of rabbit fish *Siganus fuscescens*

**DOI:** 10.1002/fsn3.2410

**Published:** 2021-07-28

**Authors:** Yi‐Teng Xia, Edwin Hok‐Chi Cheng, Brody Zhong‐Yu Zheng, Qi‐Yun Wu, Tina Ting‐Xia Dong, Ran Duan, Qi‐Wei Qin, Wen‐Xiong Wang, Karl Wah‐Keung Tsim

**Affiliations:** ^1^ Shenzhen Key Laboratory of Edible and Medicinal Bioresources HKUST Shenzhen Research Institute Shenzhen China; ^2^ Division of Life Science and Centre for Chinese Medicine The Hong Kong University of Science and Technology Hong Kong China; ^3^ Joint Laboratory of Guangdong Province and Hong Kong Region on Marine Bioresource Conservation and Exploitation College of Marine Sciences South China Agricultural University Guangzhou China; ^4^ School of Energy and Environment City University of Hong Kong Hong Kong China

**Keywords:** feed additive, growth promotion, nutrient content analysis, *Scutellaria baicalensis*

## Abstract

The root of *Scutellaria baicalensis* (Scutellaria Radix) has been used as herbal medicine for years, while its stem and leaf (aerial part) are considered as waste. The water extract from the aerial part of *S. baicalensis* (named as SBA) being included in the feeding of *Siganus fuscescens* (grey rabbit fish) has been shown to replace antibiotics in aquaculture with excellent outcome. To strengthen the usage of SBA in fish feeding, the total fish output and its nutritive value were determined here. Feeding the fishes with different doses of SBA for a month, the body length and weight were significantly increased after intake of standard feed containing 1% SBA. In parallel, the expressions of alkaline phosphatase and growth‐related factors in bone, liver, and muscle of 1% SBA‐fed fishes were markedly increased, suggesting the beneficial effects of SBA. The composition of amino acid and fatty acid in fish muscle, after intaking 1% SBA‐containing feed, was altered. In SBA‐fed fish muscle, the amounts of threonine and methionine were increased, while the amount of leucine was decreased, as compared with control group. The amounts of fatty acids, including docosahexaenoic acid, phosphatidylcholine, and phosphatidylethanolamine, were increased in the 1% SBA‐fed fish, while the amounts of triglycerides were decreased. The results indicated the growth‐promoting activity of SBA in an in vivo culture of *S. fuscescens*, as well as to increase the nutritive values of the muscle. Thus, the re‐cycle of waste products during the farming of *S. baicalensis* herb in serving as fish feeding should be encouraged.

## Graphical abstract

The SBA water extract was added into rabbit fish feeding and fed the rabbit fish in an open sea fish farm for a month. The results indicated that the feeding with 1% SBA extract showed strong growth promotion effect. The amino acid and fatty acid contents in fish muscle with SBA addition were altered. Our investigations showed the growth promotion effect of SBA extract in vivo, as well as its potential effect to alter the nutrients content in fish muscle, which further suggested the application of this waste product of S. baicalensis in fish aquaculture.

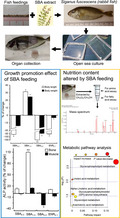

## 1 INTRODUCTION

Aquaculture is a fast‐growing industry accounting ~44% of total fish production in 2014 (Bianchi et al., 2014), but the diseases cause ~US$ 6 billion economic loss each year (Akazawa et al., 2014). About 34% of fish diseases were caused by bacteria infection (Lafferty et al., 2015). Despite the restriction in using antibiotics by policies or regulation (Lulijwa et al., 2020), tones of antibiotics, for example, enrofloxacin, oxytetracycline, and florfenicol (Liu et al., 2017), have been included in fish feeding in order to avoid bacterial infection in aquaculture. However, the abuse of antibiotics has brought many problems, for example, the increase in drug‐resistant bacteria (Wutzke et al., 2007), which becomes a core medical and social problem, worldwide. In European Union, the usage of antibiotics in animal feeding has been banned from 2006 onward (Castanon, 2007). On the other hand, the cost of feed accounts over half of total production costs in process of aquaculture (Francis et al., 2005). The finding of substitutes with characteristics of antibacterial property, stimulating growth, and cheaper cost is essential for the aquaculture industry.

Herbal products have provided an abundance of resources in searching for animal feeding additives with antibacterial and promoting growth effects (Dada, 2012). Herbal products having the characteristics of cheaper cost, eco‐friendly, minimum side effects are considered to be the ideal alternatives to replacing antibiotics in fish health management. World Health Organization (WHO) encourages the supplemented diets incorporated with medicinal herbs or plants, as to minimize the usage of synthetic chemicals in fish diet (Shakya, 2017). Herbal products have been identified in promoting fish's growth. The crude extracts of *Aloe vera*, *Carum carvi*, and *Camellia sinensis* have been reported to have a significant impact on increasing body weight, growth rate, feed intake, and feed conversion ratio in the culture of Nile tilapia (*Oreochromis niloticus*) (Gabriel, 2019). The methanol extract of green tea (*Camellia sinensis*) was shown to enhance the growth, survival rate, feeding utilization, and total protein content in black rockfish (*Sebastess chlegeli*) (Hwang et al., 2013). Garlic powder being included in the feeds showed growth promotion in Nile tilapia (Metwally, 2009) and rainbow trout (*Oncorhynchus mykiss*) (Nya & Austin, 2009).

*Siganus fuscescens*, grey rabbit fish, is wildly cultured and consumed in Southern part of China, Australia, and Indonesia (Duray, 1990). Being an excellent natural source of nutritional lipids, *S. fuscescens* is an important marine teleost fish for human consumption (Li et al., 2010). The herbivorous of *S. fuscescens* mainly consumes algae and sea grasses (Wu et al., 2020). Therefore, this feeding habitat of *S. fuscescens* is believed to be more appropriate in using herbal extract in their feeding behavior.

The root of *Scutellaria baicalensis* Georgi. (Scutellariae Radix), a traditional Chinese medicine (TCM), has long history of usage as herbal medicine with known pharmacological activities, including antivirus, antimicrobial, and anti‐inflammation (Gu et al., 2013). Chemical and pharmacological analyses have suggested that the flavonoids, that is, baicalein, baicalin, scutellarin, and wogonin, are the major active ingredients responsible for antimicrobial functions of *S. baicalensis* (Xia, Chan, et al., 2020; Xia, Hu, et al., 2020). During the production of Scutellariae Radix, the stem and leaf (aerial and non‐medicinal part) are considered as waste products. The extract from the aerial part of *S. baicalensis*, named as SBA, has been shown to substitute antibiotics in culture of *S. fuscescens* with an excellent record (Xia, Chan, et al., 2020; Xia, Hu, et al., 2020). Besides, the efficiency of antibacteria and antiinflammatory effects of SBA extract have been illustrated both in in vitro and in vivo models (Xia, Chan, et al., 2020; Xia, Hu, et al., 2020). To strengthen the usage of SBA in fish feeding, the total fish output and its nutritive value were determined here in *S. fuscescens*.

## 2 METHOD AND MATERIAL

### 2.1 Herbal preparation of SBA

The aerial part of *S. baicalensis* (SBA), collected from Hebei Province in 2017, was authenticated by Dr. Tina Dong. Voucher specimen was deposited at HKUST. The herbal preparation was reported previously (Xia, Chan, et al., 2020; Xia, Hu, et al., 2020), and the extract was dried by Labconco FreeZone Freeze Dry System. For the chemical analysis of SBA extract, a Waters 2695 series system (Waters) was employed. A Grace C18 column (particle size 5 μm, 4.60 mm × 250 mm) was used. Acetonitrile (as Solvent A) and 0.1% formic acid (as Solvent B) were the mobile phase with a flow rate of 1.0 ml/min at room temperature. Gradient elution was as follows: 0–25 min, 15%–21% of solvent A; 25–40 min, 21%–28% solvent A; 40–50 min, 28%–30% of solvent A; 50–60 min, 30%–35% solvent A; 60–65 min, 35%–15% of solvent A. A DAD detector at an absorbance of 276 nm was used.

### 2.2 Animal and experimental design

The growth‐promoting function of SBA was tested in wild‐type *S. fuscescens* fishes. The fishes were cultured in a farm in Dapeng of Shenzhen, China (22°31′N 114°28′E). Two‐week‐old fishes (8 ~ 9 cm length and body weight 8 ± 2 g) were provided by Chen Hai Seawater Products Company. Health fishes were randomly divided into 5 groups, and each one was about 20. The groupings were under standard feeding, supplemented with 0.5% SBA extract (denoted as SBA_0.5_), 1% SBA extract (denoted as SBA_1.0_), 5% SBA extract (denoted as SBA_5.0_), 0.1% enrofloxacin (denoted as ENR_0.1_), and standard feed as control. The nutrient for fish feed included the following: 30 ~ 40% protein, 6 ~ 7% lipid, 3 ~ 6% carbohydrate, 7 ~ 8% water, and 1% fish oil, purchased from Ke Lv Feeding Company. The feedings were grounded into pellets with ~2 mm diameter. The amount of feeding was 5 ~ 10% of fish body weight and fed twice a day. Every week, 4 ~ 5 fishes were collected to measure their body weight and length, then sacrificed. The tissues were collected in plastic tubes and immediately frozen at −80°C for further research. The experimental procedures were reviewed and approved by Animal Ethics Committee at the University (HKUST).

### 2.3 Alkaline phosphatase (ALP) measurement

The tissues (muscle and bone) were extracted by lysis buffer at rate of 1:10 (v/w). Lysis buffer contained 10 mM HEPES, pH 7.5, 1 mM EDTA, 1 mM EGTA, 150 mM NaCl, and 0.5% NP‐40 with the addition of the following protease inhibitors (Sigma‐Aldrich): 10 μg/ml of leupeptin, 10 μg/ml of aprotinin, and 2.5 mM benzamidine HCl. The tissue lysate was obtained by vortex for 15 min and centrifuged for 10 min at 16,000 × g at 4°C. ALP activity was measured by mixing 50 µl cell lysate with 50 µl reaction solution containing 10 mM p‐nitrophenyl phosphate (pNPP) in 0.1 M glycine (pH 10.4), 1 mM MgCl_2_, and 1 mM ZnCl_2_ at 37°C. The absorbance was measured at 405 nm after 30 min.

### 2.4 Calibration of moisture and ash

The fresh fish muscle was weighted and recorded (W1; about 1 g) and put into Eppendorf tube. The muscle samples were heated under 105°C in oven for 5 hr. Then, the weight of each tube was weighed every 30 min until the weight kept unchanged. The final weight was recorded (W2). The moisture content of fish muscle was calculated as (W1‐W2)/ W1 x 100%. The fresh muscle was weighted and recorded (W1) and put into crucible. The samples were heated under 500°C in muffle furnace for 5 hr. Then, the oven was cooled down to room temperature. The final weight was recorded (W2). The ash content of fish muscle = W2/W1 × 100%.

### 2.5 Quantitative real‐time PCR

The expression levels of several factors of growth‐related factors, including myoblast determination protein (MyoD), lipoprotein lipase (LPL), insulin‐like growth factor I (IGF‐1), insulin‐like growth factor II (IGF‐2), and growth hormone receptor (GHR), after 4 weeks of feeding, were measured by real‐time PCR. Total RNA from muscle or liver was isolated by RNAzol reagent (Molecular Research Center, Cincinnati, OH) and then reversed transcribed into cDNAs by using PrimeScript RT Reagent Kit (TakaRa Bio) reverse transcriptase, according to the manufacturer's instructions. Real‐time PCR was employed here by using FastStart Universal SYBR Green Master (ROX) according to the manufacturer's instructions (Roche Applied Science). The primers were as followed: 5′‐CCC AAC GTG TCA GAC GAG AA‐3′ (sense primer, S) and 5′‐GAG AAC ACG GGC TCC CTT C ‐3′ (antisense primer, AS) for MyoD; 5′‐CGC TCA TGT TGC AGG AAT CG ‐3′ (S) and 5′‐ACC GGC CTT TGA ATC CCA AT ‐3′ (AS) for lipoprotein lipase. Primers for growth‐related factors were as followed, 5′‐ CTG TTG TCG GTG CTG TCT CT ‐3′ (S) and 5′‐ TTG AGT TGA CGC TGG TCC TC ‐3′ (AS) for growth hormone receptor; 5′‐ ACA GCA GCC AGA CAA GAC AA ‐3′ (S) and 5′‐ CTC TCT TCC CAA GTC GCT GG ‐3′ (AS) for insulin‐like growth factor I; 5′‐ AAC TGC CTC CCA TCT TGC TC ‐3′ (S) and 5′‐ GGA GGT CAA AGA GGA CTG CC ‐3′ (AS) for insulin‐like growth factor II. β‐actin was used as an internal control, and its primer sequences were 5′‐GAG AGG GAA ATC GTG CGT GA ‐3′ (S) and 5′‐GTA GGT GGT CTC GTG GAT GC ‐3′ (AS). SYBR green signal was revealed by ABI 7500 Fast Real‐Time PCR system (Applied Biosystems). Transcript levels were quantified by using ΔCt value method, where the values of target genes were normalized by β‐actin in the same sample for comparison. PCR products were analyzed by gel electrophoresis and melting curve analysis, as to confirm the specificity.

### 2.6 Analysis of amino acid and fatty acid

The muscle samples were weighted. One hundred mg sample was extracted by 1 ml mixture of dichloromethane and methanol 2:1 (v/v). The mixture was homogenized, and then, 0.4 ml water was added and vortexed for 30 s and settled in room temperature for 10 min. The unbroken tissue was precipitated. The mixture was centrifuged at 850 × *g* under 4°C for 10 min. The clear upper layer (~0.4 ml) was moved into a new Eppendorf tube for amino acid assay, while the clear down layer (~0.4 ml) was transferred into a new glass tube. The liquid was vaporized by Termovap sample concentrator for determinations of amino acid and fatty acid. For amino acid analysis, the conditions of chromatography and mass spectrometry were described below. Waters ACQUITY UPLC HSS T3 column (2.1 × 150 mm, 1.8 μm) was applied. The mobile phase A was water with 10 mM ammonium formate and 0.1% formic acid (v/v), and B was acetonitrile. The gradient was optimized as follows: 0–2 min, 95% A; 2–4 min, 95% ~ 72% A; 4–20 min, 72% A; 20–22 min, 72% ~ 60% A; 22–35 min, 60% A; 35–45 min, 60% ~ 5% A; 45–50 min, 5% A; 50–50.1 min, 5% ~ 95% A; 50.1–55 min, 95% A. Then, PerkinElmer Mass Spectrometer Qsight 220 in MRM, POSITIVE (+) was applied, having ElectroSpray V1 Pos: 5500V; nebulizer gas: 180. For fatty acid analysis, Waters Acquity BEH C18 column (2.1 × 100 mm, 1.7 μm) was applied. The mobile phase A was water and acetonitrile at rate of 40:60 (v/v) with 5 mM ammonium formate and 0.1% formic acid (v/v), and B was isopropanol and acetonitrile at rate of 90:10 (v/v) with 5 mM ammonium formate and 0.1% formic acid (v/v). The gradient was optimized as follows: 0–13 min, 70% ~ 20% A; 13–20 min, 20% ~ 10% A; 20–21 min, 10% ~ 0% A; 21–26 min, 0% A; 26–27 min, 0% ~ 70% A; 27–30 min, 70% A; at 0.2 ml/min at 55°C. The mass spectrometer was operated in both positive and negative mode by electrospray ionization (ESI). The scan rang was from 150 m/z to 1,050 m/z.

### 2.7 Statistical analysis and other assays

The content of soluble protein in fish muscle was measured by Bradford protein assay with a kit from Bio‐Rad. Tissue lysates obtained was mixed with buffer, and absorbance was measured at 595 nm after 5min reaction. The protein content was calculated by the standard curve made by different amounts of BSA. The result was presented as the Mean ± *SEM*, calculated from 3 to 5 independent samples, with triplicated. Comparisons of the mean for untreated control cells and treated cells were analyzed using one‐way analysis of variance (ANOVA) and Student's *t* test. Significant values were represented as *, *p* < .05, **, *p* < .01. The raw data gained from mass spectrum were transformed in to excel by SCIEX OS‐Analyst software. The excel was then went through PCA and OPLS‐DA assay by SIMCA 14.1 (Umetrics, Umeå, Sweden) software. The metabolism pathway assay by Metabo Analyst 4.0 (http://www.MetaboAnalyst.ca/).

## 3 RESULT

### 3.1 The growth of *S. fuscescens* with different feedings

**FIGURE 1:**
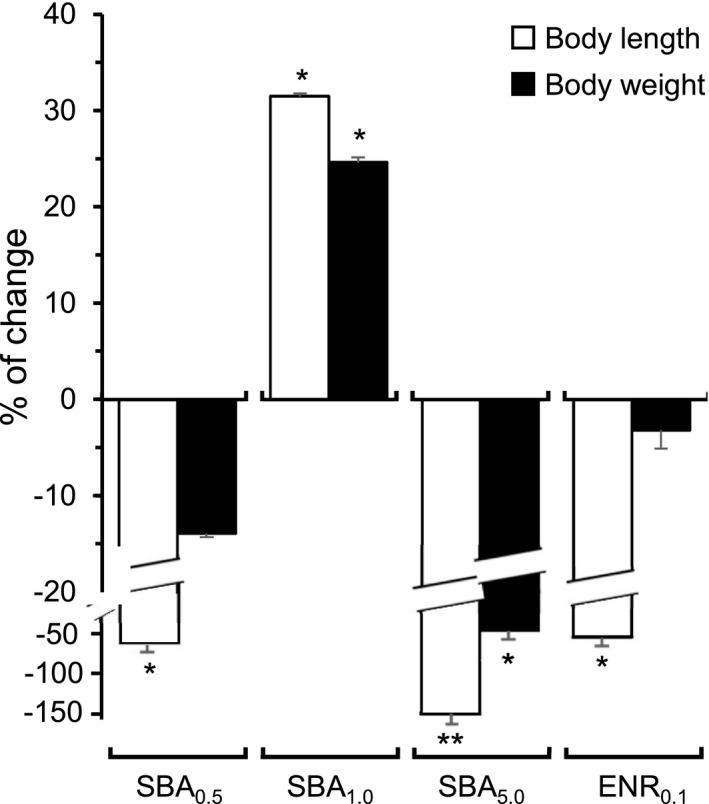
The growth performance of *S. fuscescens* after treatment. The body length and weight of *S. fuscescens* (rabbit fishes) were measured every week (see Table S1). The final measurement of 4‐week treatment was presented here. The notations are as follows: 0.5% SBA extract (SBA_0.5_), 1.0% SBA extract (SBA_1.0_), 5.0% SBA extract (SBA_5.0_), 0.1% enrofloxacin (ENR_0.1_), and control (standard feed). Values are expressed as % of change to control group (as 0, control diet), in Mean ± *SEM*, where *n* = 4–5. Statistical comparison was made with the control group; *p < .05; **p < .01

Fishes were fed with different diets for 4 weeks. The compositions of different feeding groups were, that is, 0.5% SBA extract (SBA_0.5_), 1.0% SBA extract (SBA_1.0_), 5.0% SBA extract (SBA_5.0_), 0.1% enrofloxacin (ENR_0.1_), and control (standard feed), as shown in Table 1. All fishes showed normal activity and health within the experimental period. The fish survival rate between different groups was similar. The weight and length of fishes were measured every week. The body length after feeding 4 weeks of SBA_1.0_ was significantly increased, as compared with control group, that is, an increase of ~30% in comparing to control (Figure 1). However, the fishes feeding by SBA_0.5_, SBA_5.0_, and ENR_0.1_ showed a decrease, significantly, of body length. Noticeable, the length of fish feeding with SBA_5.0_ was markedly decreased, that is, over 150%. The robust decrease in fish length of SBA_5.0_‐fed fishes could be a disturbance of feeding behavior at 5% of SBA: the high dose of SBA affected the flavor of feed, and as such the final intake was reduced. In parallel, the fish body weight showed similar trend as that in length. The intake of SBA_1.0_ caused a significant increase of ~20% in body weight, compared with control (Figure 1). Again, the body weight of fish feeding with SBA_5.0_ was significantly decreased. As a summary, the feeding of SBA_1.0_ in *S. fuscescens* was able to stimulate the overall growth.

**TABLE 1:** The composition of feeds in different groupings

Weight (%)^a^	Control^b^	SBA_0.5_	SBA_1.0_	SBA_5.0_	ENR_0.1_
Carbohydrate	45	45	45	45	45
Protein	35	35	35	35	35
Lipid	6	6	6	6	6
Cellulose	5	5	5	5	5
Water	7.9	8	7.5	7	2
Fish oil	1	1	1	1	1
SBA^c^	0	0.5	1	5	0
Enrofloxacin^d^	0	0	0	0	0.1

^a^ The content was measured by % of wet weight.

^b^ The commercial feed bought from Ke Lv feeding company.

^c^ The powder of SBA was dissolved in water at concentration of 100 mg/ml, and this solution was sprayed on the feeding particles to get the final amount.

^d^ Enrofloxacin, an antibiotic, was included in the feeding. Enrofloxacin powder was dissolved in water at concentration of 10 mg/ml, and this solution was sprayed on feeding particle to get the final amount.

### 3.2 The growth‐related factors in *S. fuscescens* with different feedings

**FIGURE 2:**
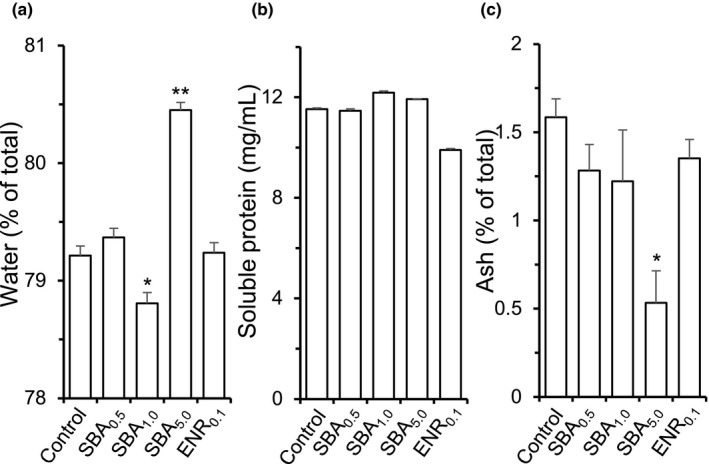
The quality of muscle after different diets. The dorsal muscle was collected from 4‐week cultured fishes. The notations are as follows: 0.5% SBA extract (SBA_0.5_), 1.0% SBA extract (SBA_1.0_), 5.0% SBA extract (SBA_5.0_), 0.1% enrofloxacin (ENR_0.1_), and control (standard feed). (a): The water content in percent of muscle wet weight. (b): The content of soluble protein in muscle in mg/m. (c): The ash content in percentage of total wet weight. Values are in Mean ± *SEM*, where *n* = 6. Statistical comparison was made with the control group; *p < .05; **p < .01

After 4 weeks of feeding in *S. fuscescens*, the muscle was collected from the dorsal part. The contents of water, soluble protein, and ash in different feeding groups were analyzed. The water content of muscle with SBA_1.0_ feeding was significantly lower than control and other groups (Figure 2a). In contrast, the water content of fish muscle, fed by SBA_5.0_, was significantly increased: this change was in line to the body weight/length under this treatment. The other groups showed no difference with the control group. The content of soluble protein did not show significant change among all experimental groups (Figure 2b). The ash content however showed a robust decrease in SBA_5.0_ feeding group, which was in line with the total body weight/length in this treatment (Figure 2c).

**FIGURE 3:**
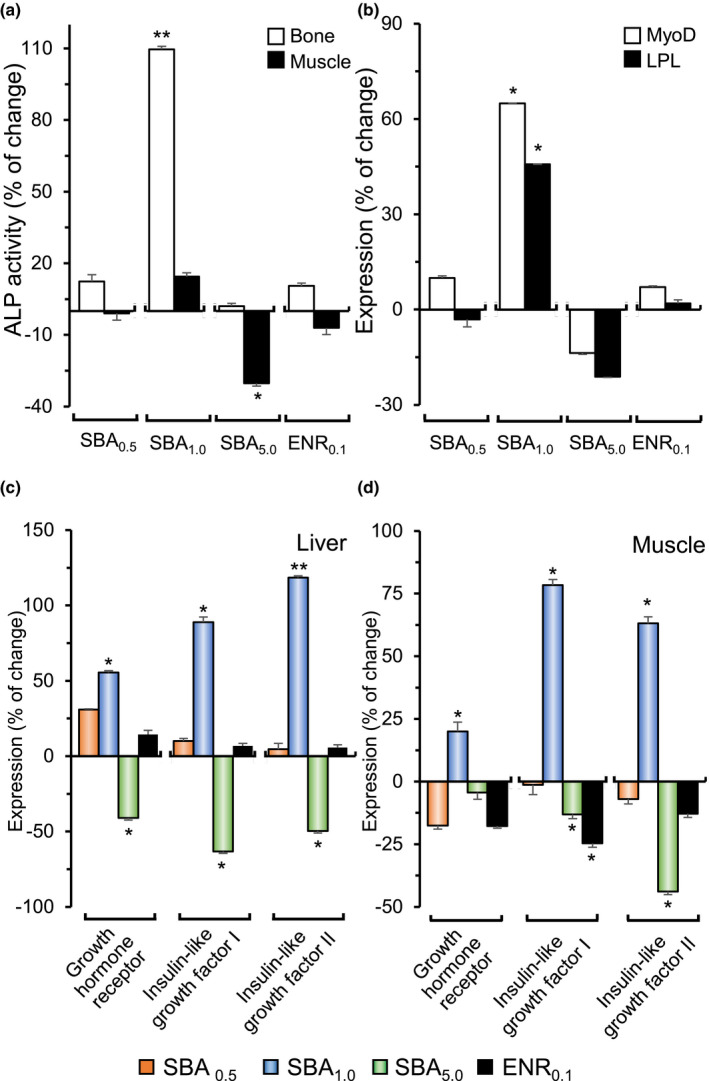
Expression of ALP, MyoD, and LPL and growth‐related factors in fish after SBA feeding. The dorsal muscle was collected from 4‐week cultured fishes. The notations are as follows: 0.5% SBA extract (SBA_0.5_), 1.0% SBA extract (SBA_1.0_), 5.0% SBA extract (SBA_5.0_), 0.1% enrofloxacin (ENR_0.1_), and control (standard feed). (a): ALP enzymatic activity in bone and muscle. (b): The expression levels of MyoD and lipoprotein lipase (LPL) in muscle. The liver and dorsal muscle were collected from 4‐week cultured fishes. The expression levels of growth hormone receptor (GHR), insulin‐like growth factor I (IGF‐I), and insulin‐like growth factor II (IGF‐II) in liver (c) and muscle (d) were measured by RT‐PCR. The values are expressed as percentage of change to control. The values are normalized to the protein amount and expressed as percentage of change to control reading (as 0, control diet), in Mean ± *SEM*, where *n* = 6. Statistical comparison was made with the control group; *p < .05; **p < .01

Alkaline phosphatase is essential for skeletal growth (Anh et al., 1998) and shows functional activities in membrane transport (Labarrère et al., 2013). Here, we analyzed ALP enzymatic activities in bone and muscle from different feeding groups. In SBA_1.0_ feeding group, the ALP activity in bone tissue was having ~110% increase, as compared to control group (Figure 3a). The increase in ALP in bone might result from increased growing activity; however, this expression of ALP was not changed significantly in muscle. In other groups, the expressions of ALP in bone and muscle did not show significant change, except the intake of SBA_5_._0_ showed a reduction in APL in muscle (Figure 3a).

During the growth in fish, MyoD regulates the activation and proliferation of satellite cell that results in formation of new muscle fibers and/or enhancement of pre‐existing fiber (Johansen & Overturf, 2005). On the other hand, lipoprotein lipase hydrolyzes triacylglycerols present in lipoproteins and provides free fatty acids for storage in adipose tissue. Lipoprotein lipase is one of the key regulatory enzymes in lipid uptake (Ma et al., 2020). Here, the total RNA was isolated from fish muscle after 4 weeks of feeding. In SBA_1.0_ feeding group, the expressions of mRNAs encoding MyoD and lipoprotein lipase in muscle were significantly increased by 65% and 45%, respectively (Figure 3b). The other groups did not show significant change in the mRNA expression, except a reduction in expression was revealed in SBA_5.0_ feeding group.

The expressions of growth hormone receptor and insulin‐like growth factor I and II in the liver and muscle were measured. The fishes, under SBA_1.0_ feeding diet, showed increases in mRNAs encoding growth hormone receptor, insulin‐like growth factor I and II in the liver and muscle (Figure 3c,d). In SBA_1.0_‐fed fishes, the mRNA expression of growth hormone receptor in liver was increased by over 50%; while the expressions of insulin‐like growth factor I and II in liver were increased by ~80% and ~110%, respectively (Figure 3c). The group having SBA_5.0_ feeding showed significant reduction in mRNAs encoding growth hormone receptor, insulin‐like growth factor I and II in liver. In muscles, the mRNA expressions of growth hormone receptor, insulin‐like growth factor I and II were significantly increased in SBA_1.0_‐fed fishes, similar to the situation in liver (Figure 3d). The muscles in other groups however showed a trend of decrease in the expression. Thus, these results further supported the growth stimulation of SBA at 1.0% inclusion in the feed.

### 3.3 Nutrition content in muscle with different feedings

To further investigate the nutritive value of *S. fuscescens* after different diets, the dorsal muscle of fishes from different groups was collected for measurements of amino acid and fatty acid. Here, total 12 kinds of amino acid were identified from fish muscle and their concentrations were determined by mass spectrum (Figure S1). According to the result, leucine was the most abundance amino acid in muscle (20.02 ± 0.05 mg/100 g), followed by alanine (13.92 ± 0.11 mg/100 g) of control *S. fuscescens* (Table S2). Some of amino acid contents in our experimental fish are similar to other reports, for example, glutamic acid having ~2.4 mg/100 g reported here versus ~1.98 mg/100 g by other (Wahyuningtyas et al., 2017). The differences in amino acid content could be caused by age, season, and life stage.

**FIGURE 4:**
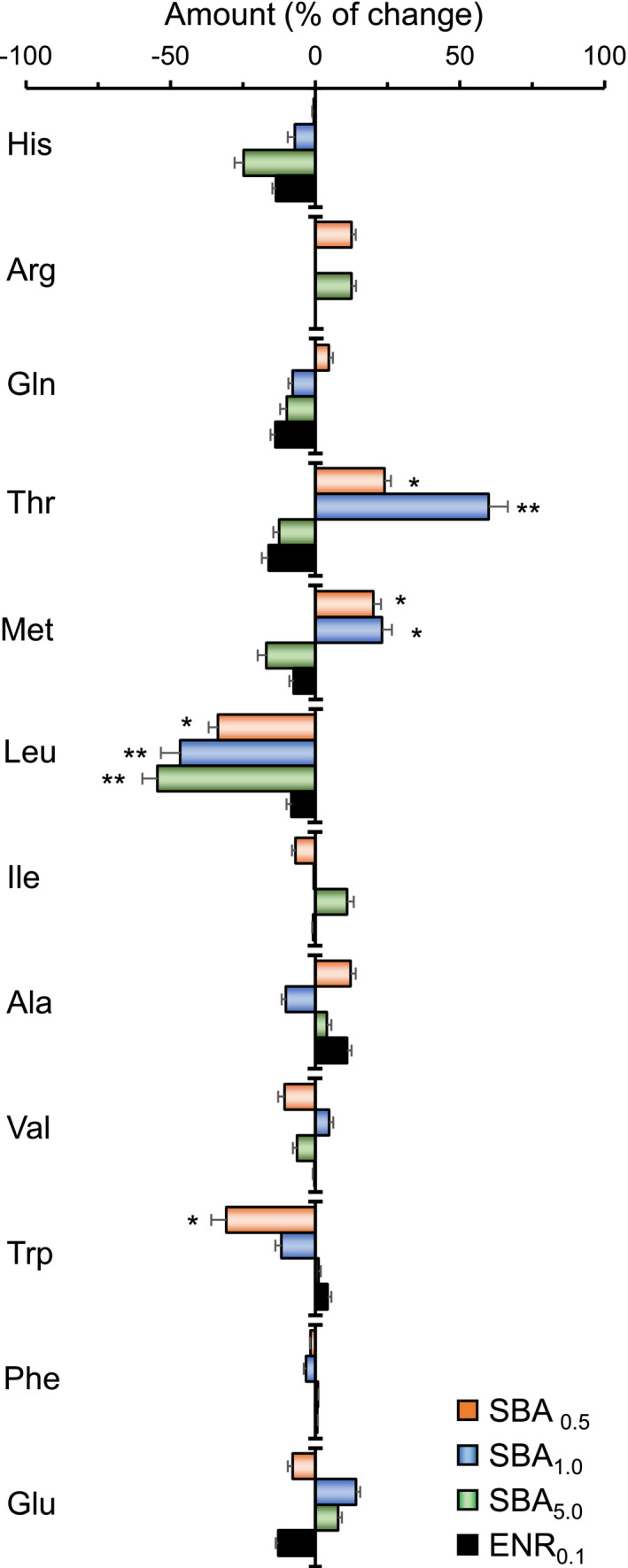
The contents of amino acid in muscle after SBA feeding. The dorsal muscle was collected from 4‐week cultured fishes. The amounts of 12 kinds of amino acids (both D, L forms) were calculated. The values are expressed as percentage of change to control reading (as 0, control diet), in Mean ± *SEM*, where *n* = 4. Statistical comparison was made with the basal group; *p < .05; **p < .01

The contents of amino acid in SBA‐fed groups were compared with control group (Figure 4). Noticeable, the amounts of threonine and methionine were significantly increased in SBA_0.5_ and SBA_1.0_ diet groups, in particular the SBA_1.0_ group. The increase in threonine in SBA_1.0_‐fed group was over 50%. The amount of leucine was decreased at least by ~40% in all three SBA‐fed groups. Here, the amino acids causing sweet taste, for example, threonine, were increased after SBA addition. The amino acid causing bitter taste, for example, leucine, was decreased. Glutamate and glutamine causing the taste of umami and sour were relatively unchanged in all groups (San Gabriel & Uneyama, 2013).

**FIGURE 5:**
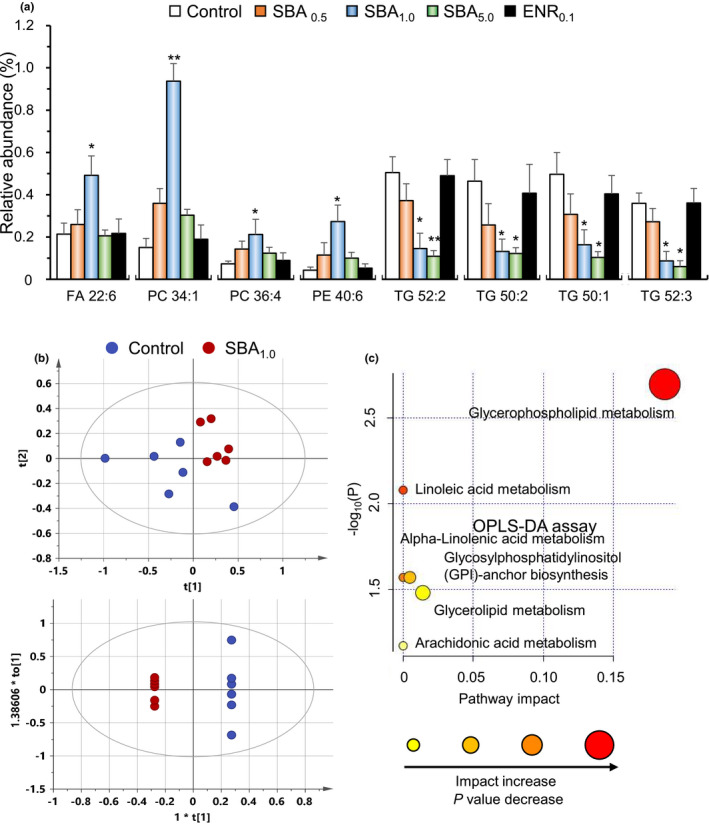
The contents of fatty acids in muscle after SBA feeding. The dorsal muscle was collected from 4‐week cultured fishes. (a): Eight fatty acids having significant difference between control group and SBA_1.0_ group were shown here. FA 22:6 (docosahexaenoic acid), PC 34:1 (1‐oleoyl‐2‐palmitoyl‐sn‐glycero‐3‐phosphocholine), PC36:4 (1,2‐di‐octadecadienoyl‐sn‐glycero‐3‐phosphocholine), and PE40:6 (1,2‐diacyl‐sn‐glycero‐3‐phosphoethanolamine). And four triglycerides including TG 52:2 (1,2‐dioctadecenoyl‐3‐hexadecanoyl‐sn‐glycerol), TG 50:2 (1‐linoleoyl‐2‐isoheptadecanoyl‐3‐isopentadecanoyl‐sn‐glycerol), TG50:1 (1,3‐dipalmitoyl‐2‐oleoylglycerol), and TG 52:3 (1‐palmitoyl‐2‐linoleoyl‐3‐oleoyl‐sn‐glycerol). The values are expressed as the relevant amount, in Mean ± *SEM*, where *n* = 4. Statistical comparison was made with the control group; *p < .05; **p < .01. (b): The principal components analysis (PCA; upper panel) and orthogonal projections to latent structures discriminant analysis (OPLS‐DA; lower panel) on MS result from control diet and SBA_1.0_ diet, where *n* = 6. (c): Metabolic pathway analysis by using MetaboAnalyst 4.0. The *x*‐axis represents the pathway impact value computed from pathway topological analysis, and the *y*‐axis is the log of the *p*‐value obtained from pathway enrichment analysis. The glycerophospholipid metabolism pathway shows the most significantly change with both a high‐log(*p*) value and high impact value

To analyze the alteration of fatty acid, induced by SBA feeding, the method of LC‐MS was applied here. The distinction of fatty acid profiles between control diet and SBA_1.0_ diet was obvious. The fatty acids having variable importance projection (VIP) index over 1 and absolute value of partial correlation coefficient over 0.4 were identified and chosen for analysis (Table S3a). The fatty acid having *p* value below 0.05 indicated the significant change, as compared to control diet. The identities and molecular structures of these fatty acids were revealed (Table S3b). Here, eight fatty acids with most significant difference in content were analyzed. Four fatty acids were significantly increased in SBA_1.0_‐fed group, including FA 22:6 (docosahexaenoic acid), PC 34:1 (1‐oleoyl‐2‐palmitoyl‐sn‐glycero‐3‐phosphocholine), PC36:4 (1,2‐di‐octadecadienoyl‐sn‐glycero‐3‐phosphocholine), and PE40:6 (1,2‐diacyl‐sn‐glycero‐3‐phosphoethanolamine) (Figure 5a). The SBA_1.0_ diet significantly increased docosahexaenoic acid by over twofold, as compared to control diet. In addition, phosphatidylcholines and phosphatidylethanolamine showed significant increase from twofold to fivefold (Figure 5a). In contrast, four triglycerides were significantly decreased in the SBA_1.0_‐fed group, as well as SBA_5.0_‐feeded group, including TG 52:2 (1,2‐dioctadecenoyl‐3‐hexadecanoyl‐sn‐glycerol), TG 50:2 (1‐linoleoyl‐2‐isoheptadecanoyl‐3‐isopentadecanoyl‐sn‐glycerol) TG50:1 (1,3‐dipalmitoyl‐2‐oleoyl‐glycerol), and TG 52:3 (1‐palmitoyl‐2‐linoleoyl‐3‐oleoyl‐sn‐glycerol). The decreases were at least over threefold.

The data generated from mass spectrum were subjected to PCA analysis by SIMCA 14.1 (Umetrics, Umeå, Sweden) software. The distinction between control diet and SBA_1.0_ diet was obvious on a PCA score plot having variation of the samples (Figure 5b). The values of R2X and Q2 were 67.5% and 47.6%, respectively, meaning a significant change in fatty acid content after SBA feeding. Moreover, OPLS‐DA model was applied to the analysis of metabolomics data. The score plot showed a clear separation between control group and SBA_1.0_ group (Figure 5b). The model parameters of R2X, R2Y and Q2 were 84.6%, 100%, and 79.0%, respectively, in the positive pattern. By having Metabo Analyst 4.0 on the metabolic pathway analysis, the SBA_1.0_ diet was shown to affect the glycerophospholipid metabolism pathway at a higher impact (Figure 5c), which could account the observed change in fatty acid profiling.

## 4 DISCUSSION

The standard feeds using here contained 30 ~ 40% protein, 6 ~ 7% lipid, 3 ~ 6% carbohydrate, 7 ~ 8% water, and 1% fish oil. This formulation of feed is rather similar to the commercial source ARASCO (Yousif et al., 2005). Having the inclusion of SBA at 1% in the fish feeding, the growth of body length and weight of *S. fuscescens* was markedly increased. The results strongly suggest the beneficial application of SBA in aquaculture, which includes the following: (a) serving as a substitute for antibiotics (Xia, Chan, et al., 2020; Xia, Hu, et al., 2020); (b) suppressing pro‐inflammatory cytokines in bacteria‐contaminated environment (Xia, Chan, et al., 2020; Xia, Hu, et al., 2020); (c) serving as part of the feeding nutrient that stimulating total yield; and (d) re‐cycle of waste product from Chinese herb farming in reducing the cost for aquaculture. In contrast, the overdose of SBA, that is, 5%, in the feed caused a reduction in total yield. These results are in line with inclusion of herbal extracts from roots of *S. baicalensis* in feeding olive flounder (*Paralichthys olivaceus*), which has demonstrated that the 2% of root extract could able to stimulate growth; but 3% to 5% of the root extract contrary could suppress the growth (Cho et al., 2013). In addition, the 2% herbal extract deriving from a mixture of *S. baicalensis* root and *Poria cocos* in feeding redfish (*Sciaenops ocellatus)* caused an increase of 10 to 30% growth (Ma et al., 2009).

The water content of muscle in SBA_1.0_‐fed fishes was lower than that of control feeding group; while SBA_5.0_‐fed fishes showed higher content. These values of water content in fish muscles were in line to other reports (Osako et al., 2003), except our result was slightly higher. The discrepancy could be accounted by whole rabbit fish of much bigger size being used in other report (Osako et al., 2005). The lower value of water content in the muscle has been identified in better growth of Nile tilapia (Ayisi et al., 2017) and carp (*Cyprinus carpio*) (Daudpota, 2020). An increase in ALP activity in bone is corresponding to an increase in total growth (Xie et al., 2011). Indeed, an increased ALP activity during fish growth was reported in Japanese sturgeon (*Acipenser schrenckii*) and grass carp (*Ctenopharyngodon idella*) (Li et al., 2016; Xu et al., 2012). In SBA_1.0_‐fed group, the ALP activity in bone tissue was about double of that of control group, which could account the increased growth. In contrast, the feeding by SBA_5.0_ caused a decrease in body size/weight, as well as other growth‐related parameters. This high‐dose effect of SBA could be affected the feeding disturbance, that is, eating less by fish.

Myoblast determination protein, a muscle transcriptional activator, is directing formation of new muscle fiber and enhancing pre‐existing fiber (Johansen & Overturf, 2005). The expression of MyoD in muscle was positive correlated with growth, as demonstrated in grass carp (Zhao et al., 2018) and zebra fish (Ulloa et al., 2013). Another biomarker sensitive to SBA feeding is lipoprotein lipase, an enzyme hydrolyzing triacylglycerols to free fatty acids (Zheng et al., 2010), which is a key regulatory enzyme in lipid uptake and energy storage (Tian et al., 2013). In fish feeding, a high dietary lipid in feeds could significantly increase the expression of lipoprotein lipase in the liver and muscle (Han et al., 2011; Zheng et al., 2017). In addition, we have shown the expressions of growth hormone receptor and insulin‐like growth factor I and II in the liver and muscle are increased in fish feeding with SBA_1.0_. The correlation of these growth‐related factors has been shown to play a significant role in fish growth (Carnevali et al., 2005; Kutluyer et al., 2017; Peterson et al., 2005; Wu et al., 2019). Baicalein, a flavonoid found in SBA extract, has been proposed to stimulate the growth of fish in culture (Liang et al., 2012).

Fish muscle is an important dietary source of animal proteins and amino acids (Mohanty et al., 2014). Besides, the content of amino acid affects the taste and flavor of fish meat (Gunlu & Gunlu, 2014). According to our result, the amount of soluble protein in fish muscle from different diets feeding groups was unchanged; however, the content of individual amino acids in muscle showed differences. The abundance of threonine and methionine, the amino acids causing the sweet taste, was significantly increased in SBA‐fed groups, while the abundance of leucine, amino acid causing the bitter taste, was decreased in all three SBA diet groups. Besides, glutamate and glutamine causing the taste of umami and sour were almost kept unchanged in our experiment (San Gabriel & Uneyama, 2013). This change in amino acid content may improve the taste of fish.

Fatty acids have vital roles as a source of membrane constituents, energy storage, and metabolic and signaling mediators (San Gabriel & Uneyama, 2013). Here, we found that the contents of fatty acids in fish muscle were altered after feeding with SBA‐containing feed. The amounts of docosahexaenoic acid, phosphatidylcholine, and phosphatidylethanolamine were increased in SBA‐fed fish muscles. Docosahexaenoic acid (Mohanty et al.) is a long‐chain highly unsaturated omega‐3 fatty acid, which is essential for the growth and development of the brain in infants (Horrocks & Yeo, 1999), as well as possible treatment for Alzheimer's disease (Cunnane et al., 2009). Few triglycerides were significantly decreased, including 1,2‐dioctadecenoyl‐3‐hexadecanoyl‐sn‐glycerol, 1‐linoleoyl‐2‐isoheptadecanoyl‐3‐isopentadecanoyl‐sn‐glycerol, 1,3‐dipalmitoyl‐2‐oleoyl‐glycerol, and 1‐palmitoyl‐2‐linoleoyl‐3‐oleoyl‐sn‐glycerol, in SBA‐fed fish muscle. This result is similar to the intake of *S. baicalensis* herbal extract in mice (Song et al., 2013) and chicken (Króliczewska et al., 2004). Scutellarin (Lu et al., 2013) and baicalin (Guo et al., 2009), two major flavonoids in *S. baicalensis,* were proposed to contribute to this outcome via PPARγ, C/EBPα, and AMPK pathways in regulating the glycerophospholipid metabolism. This indication is supported by the pathway analysis as reported here.

## 5 CONCLUSION

The inclusion of herbal extract of aerial part of *S. baicalensis* in fish feeding shows benefits for the growth of rabbit fish. The growth performance and the expression of growth‐related proteins, as well as nutrition contents in muscle, are significantly enhanced by appropriate diet. Our results indicate that the diet containing 1% SBA herbal extract in the feed could be a foundation for its large‐scale cultivation of fish. For an economic reason, the waste product during the farming of *S. baicalensis* can be applied as a feeding additive to promote the fish's growth as well as alter the nutrition contents of fish meat.

## CONFLICT OF INTEREST

The authors declare that there are no conflicts of interest.

## AUTHOR CONTRIBUTIONS

**Yi‐Teng Xia:** Data curation (lead) ; Investigation (lead) ; Writing‐original draft (lead) ; Writing‐review & editing (equal) . **Edwin Hok‐Chi Cheng:** Data curation (equal) ; Investigation (equal) ; Methodology (equal) . **Brody Zhong‐Yu Zheng:** Data curation (equal) ; Investigation‐Supporting; Methodology (equal) . **Qi‐Yun Wu:** Methodology‐Supporting; Software (equal) ; Writing‐review &editing‐Supporting. **Tina Ting‐Xia Dong:** Funding acquisition (equal) ; Methodology‐Supporting; Project administration (equal) ; Resources (lead) . **Ran Duan:** Data curation‐Supporting; Methodology‐Supporting; Resources (equal) . **Qi‐Wei Qin:** Funding acquisition (equal) ; Project administration (equal) ; Resources‐Supporting. **Wen‐Xiong Wang:** Conceptualization (equal) ; Funding acquisition‐Supporting; Project administration‐Supporting; Supervision‐Supporting. **Karl W. K. Tsim:** Funding acquisition (lead) ; Project administration (lead) ; Supervision (lead) ; Writing‐review & editing (lead)

## Supporting information

App S1

## Data Availability

Research data are not shared.
