# Supplementary material for: Feeding containing the aerial part of Scutellaria baicalensis promotes the growth and nutritive value of rabbit fish Siganus fuscescens
Source: Food Sci Nutr. 2021 Jul 28;9(9):4827–38. doi: 10.1002/fsn3.2410 (PMC8441375; doi:10.1002/fsn3.2410)
Supplement: Supplementary file 1 — App S1 [file FSN3-9-4827-s001.docx]

**Supplementary Table 1. The measurements of fish length and weight during the 4 weeks of study.**

Body length (cm)

| week | Control | SBA_0.5_ | SBA_1.0_ | SBA_5.0_ | ENR_0.1_ |
| --- | --- | --- | --- | --- | --- |
| 0 | 9.00±0.01 |  |  |  |  |
| 1 | 9.50±0.11 | 9.60±0.10 | 8.92±0.05 | 9.68±0.08 | 9.72±0.04 |
| 2 | 8.82±0.14 | 9.82±0.04 | 9.72±0.05 | 9.32±0.05 | 10.12±0.18 |
| 3 | 8.50±0.05 | 8.78±0.16 | 8.68±0.18 | 8.88±0.11 | 9.92±0.10 |
| 4 | 9.88±0.05 | 9.32±0.10 | 10.15±0.27^*^ | 8.82±0.03^**^ | 9.40±0.06 |

Body weight (g)

| week | Control | SBA_0.5_ | SBA_1.0_ | SBA_5.0_ | ENR_0.1_ |
| --- | --- | --- | --- | --- | --- |
| 0 | 8.57±0.17 |  |  |  |  |
| 1 | 11.06±0.28 | 12.16±0.33 | 11.92±0.18 | 9.69±0.16 | 9.56±0.09 |
| 2 | 10.20±0.19 | 13.01±0.15 | 12.26±0.11 | 11.66±0.22 | 15.02±0.43^**^ |
| 3 | 12.28±0.11 | 9.92±0.19 | 12.12±0.14 | 13.12±0.28 | 13.97±0.27 |
| 4 | 13.38±0.04 | 12.71±0.21 | 14.56±0.13^*^ | 10.96±0.08^**^ | 13.22±0.10 |

The dorsal muscle was collected from 4-week cultured fishes. The notations are as follows: 0.5% SBA extract (SBA_0.5_), 1.0% SBA extract (SBA_1.0_), 5.0% SBA extract (SBA_5.0_), 0.1% enrofloxacin (ENR_0.1_), and control (standard feed). The values are in Mean ± SEM, where *n* = 4; *p* < 0.05; ** *p* < 0.01.

**Supplementary Table 2. The contents of amino acids in muscle of fish after SBA feeding.**

| Amino acid  (mg/100g^a^) | Control | SBA_0.5_ | SBA_1.0_ | SBA_5.0_ | ENR_0.1_ |
| --- | --- | --- | --- | --- | --- |
| Histidine | 1.69±0.11 | 1.68±0.32 | 1.57±0.44 | 1.27±0.17 | 1.46±0.17 |
| Arginine | 0.08±0.01 | 0.09±0.01 | 0.08±0.01 | 0.09±0.02 | 0.08±0.01 |
| Glutamine | 1.52±0.56 | 1.59±0.09 | 1.40±0.14 | 1.37±0.06 | 1.31±0.82 |
| Threonine | 1.67±0.21 | 2.07±0.11 | 2.67±0.08 | 1.46±0.22 | 1.40±0.12 |
| Methionine | 2.95±0.35 | 3.54±0.0.27 | 3.63±0.14 | 2.45±0.74 | 2.73±0.21 |
| Leucine | 20.04±0.05 | 13.30±0.13 | 10.67±0.09 | 9.10±0.12 | 18.39±0.05 |
| Isoleucine | 11.32±0.09 | 10.54±0.10 | 11.26±0.03 | 12.56±0.08 | 11.24±0.05 |
| Alanine | 13.92±0.11 | 15.62±0.09 | 12.50±0.14 | 14.48±0.12 | 15.45±0.04 |
| Valine | 3.18±0.14 | 2.84±0.11 | 3.33±0.12 | 2.98±0.08 | 3.17±0.18 |
| Tryptophan | 0.94±0.20 | 0.65±0.51 | 0.83±0.36 | 0.95±0.33 | 0.98±0.10 |
| Phenylalanine | 9.64±0.19 | 9.48±0.12 | 9.33±0.08 | 9.72±0.09 | 9.71±0.04 |
| Glutamate | 2.42±0.02 | 2.23±0.13 | 2.76±0.10 | 2.61±0.12 | 2.11±0.15 |

The dorsal muscle was collected from 4-week cultured fishes. The notations are as follows: 0.5% SBA extract (SBA_0.5_), 1.0% SBA extract (SBA_1.0_), 5.0% SBA extract (SBA_5.0_), 0.1% enrofloxacin (ENR_0.1_), and control (standard feed). The values are in Mean ± SEM, where *n* = 4; *p* < 0.05; ** *p* < 0.01.

^a^ Amount of amino acid (D and L forms) was calculated by mg/100g of wet weight.

**Supplementary Table 3. Distinct fatty acids identified in SBA-fed fish muscle.**

A

| Fatty acid | Name | VIP | *p*(corr) | *p* value |
| --- | --- | --- | --- | --- |
| FA 22:6 | Docosahexaenoic acid | 1.80 | -0.76 | 0.041 |
| PC 34:1 | 1-oleoyl-2-palmitoyl-sn-glycero-3-phosphocholine | 2.10 | -0.71 | 0.009 |
| PC 36:4 | 1,2-di-octadecadienoyl-sn-glycero-3-phosphocholine | 1.01 | -0.81 | 0.041 |
| PE 40:6 | 1,2-diacyl-sn-glycero-3-phosphoethanolamine | 1.22 | -0.7 | 0.002 |
| TG 52:2 | 1,2-dioctadecenoyl-3-hexadecanoyl-sn-glycerol | 5.56 | 0.68 | 0.015 |
| TG 50:2 | 1-linoleoyl-2-isoheptadecanoyl-3-isopentadecanoyl-sn-glycerol | 5.40 | 0.67 | 0.015 |
| TG 50:1 | 1,3-dipalmitoyl-2-oleoylglycerol | 5.11 | 0.75 | 0.015 |
| TG 52:3 | 1-palmitoyl-2-linoleoyl-3-oleoyl-sn-glycerol | 4.91 | 0.66 | 0.015 |

B

| No. | |  | 1 | 2 | 3 | 4 | 5 | 6 | 7 | 8 |
| --- | --- | --- | --- | --- | --- | --- | --- | --- | --- | --- |
| Identification | |  | FA 22:6 | PC 34:1 | PC 36:4 | PE 40:6 | TG 52:2 | TG 50:2 | TG 50:1 | TG 52:3 |
| tR (min) | |  | 6.22 | 13.3 | 12.22 | 13.27 | 23.05 | 22.18 | 23.1 | 22.26 |
| Formula | |  | C_22_H_32_O_2_ | C_42_H_82_NO_8_P | C_44_H_80_NO_8_P | C_45_H_78_NO_8_P | C_55_H_102_O_6_ | C_53_H_98_O_6_ | C_53_H_100_O_6_ | C_55_H_100_O_6_ |
| Chain |  |  | 22:06 | 16:0  /18:1 | 16:0  /20:4 | 18:0  /22:6 | 16:0  /18:1/18:1 | 16:1  /16:0/18:1 | 16:0  /16:0/18:1 | 16:1  /18:2/18:0 |
| POS | ESI+ |  | - | [M+H]^+^ | [M+H]^+^ | [M+H]^+^ | [M+NH_4_]^+^ | [M+NH_4_]^+^ | [M+NH_4_]^+^ | [M+NH_4_]^+^ |
|  | m/z |  | - | 760.585 | 782.570 | 792.553 | 876.801 | 848.769 | 850.785 | 874.785 |
|  | Cal m/z |  | - | 760.585 | 782.569 | 792.554 | 876.802 | 848.770 | 850.786 | 874.786 |
|  | Diff (ppm) |  | - | -0.4 | 1.4 | -0.8 | -1 | -1.3 | -1.3 | -0.6 |
|  | Fragment ions (m/z) |  | - | 184 | 184 | 651 | 603, 577 | 577,  575, 549 | 577, 551 | 603, 601, 577, 575 |
| NEG | ESI- |  | [M-H]^-^ | [M+FA-H]^-^ | [M+FA-H]^-^ | [M-H]^-^ | - | - | - | - |
|  | m/z |  | 327.234 | 804.583 | 826.563 | 790.543 | - | - | - | - |
|  | Cal m/z |  | 327.233 | 804.576 | 826.560 | 790.539 | - | - | - | - |
|  | Diff (ppm) |  | 2.8 | 8.5 | 3.3 | 4.8 | - | - | - | - |
|  | Fragment ions (m/z) |  | 283,  242, 229 | 744,  255, 281 | 480,  303, 255 | 327, 283 | - | - | - | - |

**(A):** The molecular formula, name, variable importance in projection (VIP) value, partial correlation coefficient (*p*corr) value, and calculated *p* value through Mann–Whitney assay of fatty acid, showing significantly different between control diet and SBA_1.0_ diet. **(B):** The identification of fatty acids from mass spectrum result.

**Supplementary Figure**. The monitored MRM parameters of identified amino acids (upper panel). A representative ion chromatogram of TSPC-labeled D, L-amino acids. Electrospray V1 Pos: 5500V; nebulizer gas: 180 (lower panel).
